# Interaction between Phosphate Solubilizing Bacteria and Arbuscular Mycorrhizal Fungi on Growth Promotion and Tuber Inulin Content of *Helianthus tuberosus* L

**DOI:** 10.1038/s41598-020-61846-x

**Published:** 2020-03-18

**Authors:** Sabaiporn Nacoon, Sanun Jogloy, Nuntavun Riddech, Wiyada Mongkolthanaruk, Thomas W. Kuyper, Sophon Boonlue

**Affiliations:** 1 Department of Microbiology, Faculty of Science, Khon Kaen University, Khon Kaen, 40002 Thailand; 2 Department of Agronomy, Faculty of Agriculture, Khon Kaen University, Khon Kaen, 40002 Thailand; 3 Soil Biology Group, Wageningen University & Research, P.O. Box 47, 6700 AA Wageningen, The Netherlands

**Keywords:** Applied microbiology, Soil microbiology

## Abstract

Arbuscular mycorrhizal fungi (AMF) and phosphate solubilizing bacteria (PSB) could interact synergistically because PSB solubilize sparingly available phosphorous compounds into orthophosphate that AMF can absorb and transport to the host plant. Little is known about the interactions between these two groups in terms of promoting Jerusalem artichoke, *Helianthus tuberosus* L., which is widely planted by farmers because of its high inulin content. Production depends mainly on synthetic fertilizers as source of plant nutrients. This study aimed to isolate and characterize PSB and investigate the effects of co-inoculation of AMF and PSB on plant performance and inulin accumulation. Isolate UDJA102x89-9, identified as *Klebsiella variicola* (KV), showed phosphate-solubilizing ability and produced high amounts of several organic acids *in vitro* and of indole-3-acetic acid (IAA). The experiment combined KV and two AMF species (*Glomus multisubtensum* (GM) and *Rhizophagus intraradices* (RI)). Co-inoculation of KV with RI, in combination with rock phosphate, showed the largest increases in plant growth and tuber inulin content, compared both to an unfertilized and fertilized control. This result would reveal whether the phosphate solubilization and IAA property of the PSB *in vitro* played a significant role in changing plant growth and production, and the available P was subsequently taken up and transported to plant roots by AMF. The high combined effect may have the potential for use by farmers in the future as a biofertilizer for inulin production by *Helianthus tuberosus* L.

## Introduction

Jerusalem artichoke (*Helianthus tuberosus* L.) is an economically important plant mainly due to the high application potential of its tubers in the bioenergy (biofuel) sector and the uses of leaves and stems in the pharmaceutical sector, because of its bioactive compounds^1,2^. The roots of Jerusalem artichoke have been explored particularly for the production of functional-food ingredients such as inulin, oligofructose and fructose^3^. A number of valuable bioactive compounds can be used for antifungal, antioxidant and anticancer activities^4^. At present, Jerusalem artichoke is increasingly grown as a specialty plant by many farmers because of increased revenues. In a short time period, the plant has become very popular for cultivation. However, high productivity of Jerusalem artichoke depends mainly on synthetic fertilizers as a source of plant nutrients. This practice not only increases production costs, but potentially causes environmental pollution. The use of phosphorus (P) fertilizer is also relatively inefficient, because of the strong sorption to and fixation by metal oxides in the soil matrix. As a result, 95–99% of soil P occurs as insolubilized, immobilized, and/or precipitated forms that cannot be immediately utilized by plants^5^. Plant growth-promoting microorganisms present in the soil employ strategies to scavenge and mine the soil and make P available to plants.

Arbuscular mycorrhizal fungi (AMF) have a symbiotic relationship with the roots of approximately 80% of plant species and this symbiosis increases P and micronutrient uptake and thereby promote the growth of their host plant^6^. AMF provide nutrients to their host plants by producing hyphae that grow out from plant roots, effectively increasing the soil volume from which immobile nutrients can be acquired. Most agricultural crops perform better and are more productive when they are colonized by AMF compared with non-mycorrhizal plants. Apart from beneficial effects on plant biomass, AMF also frequently increases plant nutrient concentrations and improves the amounts of secondary compounds in plants^7^.

Phosphate solubilizing bacteria (PSB) form an integral component of the phosphorus soil cycle. They convert insoluble, inorganic and organic P forms into the bioavailable orthophosphate form which is the only form that can be taken up by plant roots^8^. PSB release several organic acids, including citric, oxalic, fumaric, malic, formic, lactic, and succinic acid. These organic acids can reduce the pH of surrounding soils and contribute to solubilizing phosphate in the rhizosphere^9,10^. In addition, PSB can produce Indole acetic acid (IAA), which stimulates the production of longer roots and increases the number of root hairs and root laterals^11^. Mohite *et al*. reported that IAA-producing bacteria are efficient as biofertilizer inoculants in promoting plant growth^12^.

Several reports documented that PSB isolates that produce IAA could improve vegetative growth parameters, photosynthesis, and NPK concentrations in plants with significant improvements over the control^13^.

An *in vitro* trial and a greenhouse trial found that either strains of PSB or AMF had the capacity to improve plant P acquisition. It also noted that dual inoculation of these two microorganisms stimulated plant growth to a larger extent than inoculation with either microorganism alone under sterile soil conditions^14^. Such dual-inoculated plants showed increased yield and had higher N and P concentration of plant tissues than single-inoculated plants^15^. A plausible mechanism for interactive (synergistic) effects is that PSB solubilize P and increase P availability, which subsequently is taken up by the AMF and delivered to the plant^16^. However, there is limited information on possible synergistic effects of AMF and PSB in nonsterile soil condition, where competition with the indigenous microbial community may modify the outcome of such interactions. We therefore characterized PSB that were isolated from the rhizosphere of Jerusalem artichoke variety HEL65 and evaluated in the field the possible synergistic effect of PSB and AMF on growth promotion. We focused both on general effects on plant performance and specifically on possible enhancement of tuber inulin content, where such synergistic effects have not been reported before.

## Materials and methods

### Rhizosphere soil sampling

Rhizosphere soil samples (soil adhering to the roots) were collected from Jerusalem artichoke variety HEL65 field-grown plants at seven farms in the central and northeastern regions of Thailand, from Khon Kaen, Nakhon Ratchasima, Udon Thani, Phetchabun and Saraburi provinces. Soil samples were taken at a depth of 0 to 15 cm. In each plot three samples were taken from the center. Each sample was individually placed in an aseptic plastic bag and immediately stored in a cooler until arrival at the laboratory. The samples were stored at 4 °C until isolation of the PSB.

### PSB isolation

The microbes in the 21 rhizosphere samples were tested for their ability to solubilize P using the dilution plate count technique on Pikovskaya’s agar (PKV) medium containing 5% tri-calcium phosphate at 30 °C for 72 h. PSB isolates were identified by a halo zone around their colonies. Colonies that showed larger solubilization (as assessed by a larger halo zone) were further purified. Pure colonies were spot-inoculated on PKV agar medium and incubated for 72 h at 30 °C. Phosphate solubilization potency was measured based on the Phosphate Solubilization Index (PSI) as described by Morales *et al*.^17^, which was calculated using the following equation.

1 $${\rm{PSI}}=\frac{{\rm{Total}}\,{\rm{diameter}}\,{\rm{of}}\,{\rm{halo}}\,{\rm{zone}}}{{\rm{Colony}}\,{\rm{diameter}}}$$

### Estimation of soluble phosphorus and indole 3 acetic acid in culture broth

Eleven selected bacterial strains were evaluated for P-solubilizing activity in PKV liquid medium containing 0.5% tri-calcium phosphate. PSB strains were grown in the broth medium and shaken at 150 rpm at 30 °C for 72 h. The pH value of the medium was then measured. The cultured broth medium was centrifuged at 8,000 rpm at 4 °C for 15 min to remove bacterial cells. The amount of released soluble phosphate was determined by the Vanadate-molybdate yellow method^18^ by using a spectrophotometer at a wavelength of 420 nm. In addition, the types of organic acid in the medium were analyzed by HPLC, which was slightly modified from the method described by Surapat *et al*.^19^. PSB were inoculated into Pikovskaya’s medium by shaking conditions operated at 150 rpm at 30 °C for 72 h. The cultured broth medium was centrifuged for 20 min at 8,000 rpm at 4 °C in a Herttich Rotina 35 R to remove bacterial biomass. The supernatant was filtered through a 0.2 µm filter, and 20 µL of filtrate was then injected into an HPLC column LC-20AD (Shimadzu, Kyoto, Japan) equipped with a SPD-M20A diode array detector. Organic acid separation was carried out on an Inertsil ODS-3 column (GL Sciences, Tokyo, Japan) with 50 mM K_2_HPO_4_ in phosphoric acid (pH 2.7) as a mobile phase. The retention time was recorded at a wavelength of 210 nm. The organic acids in the supernatant were identified by their retention time, corresponding with their standards. To determine the amount of IAA production, a colorimetric technique was undertaken with Van Urk Salkowski’s reagent^20^.

### Identification of PSB isolates

Identification of those 11 PSB isolates was carried out by analysis of 16 s rDNA sequences. Genomic DNA was extracted by the freeze-thaw method and amplified using PCR amplification. The universal primers 1525 R (5′-AAAGGAGGTGATCCAGCC-3′) and 27 F (5′-AGAGTTTGATCCTGGCTCAG-3′) were used for amplification. The reaction conditions included an initial denaturation of 1 min at 94 °C, followed by 30 cycles of denaturation at 94 °C for 30 s, annealing at 55 °C for 30 s, and extension at 72 °C for 30 s. A final extension at 72 °C for 10 mins was undertaken at the end of the amplification. PCR products were sequenced externally by First BASE Laboratories Sdn Bhd, Malaysia. The DNA sequence was submitted to GenBank of the DNA Data Bank of Japan (DDBJ) for homology analysis using the BLASTN program.

### Preparation of PSB and AMF inoculum

Bacterium isolate UDJA102x89-9 identified as *Klebsiella variicola* (KV) was selected for subsequent pot trials because this isolate showed the highest IAA production and had a high phosphate solubilization ability. The bacterial starter was prepared by incubation in the nutrient broth (NB) at 150 rpm at 30 °C for 72 h and the cells were harvested by centrifugation at 8,000 rpm at 4 °C for 20 min. The cell pellet was resuspended in sterile distilled water and made up to a final concentration of 10^9^ CFU mL^−1^. Two strains of AMF, *Glomus multisubtensum* KKU-UD-JA-DBr (GM), and *Rhizophagus intraradices* KKU-Wh (RI)) were obtained from the stock soil inoculum in the Mycotechnology laboratory at the Department of Microbiology, Khon Kaen University. Both strains were previously shown to be highly effective in growth promotion of Jerusalem artichoke in pot trials (data not shown). Each AMF species was multiplied by a pot culture technique using maize as a host plant^21^.

### Pot trial experiment

A pot experiment was conducted in the dry season between February and May 2015 in an open-sided greenhouse at Khon Kaen University’s agronomy farm in Khon Kaen, Thailand (16° 28′N, 102° 48′E, 200 meters above mean sea level). A sandy loam soil was used which had the following chemical properties: pH 5.27, 3.9 g soil organic matter kg^−1^, 160 mg total N kg^−1^, 50 mg total P kg^−1^, 280 mg total K kg^−1^, 5 mg available P kg^−1^, 36 mg exchangeable K kg^−1^, 125 mg exchangeable Ca kg^−1^ and 56 mg exchangeable Na kg^−1^.

The pots were arranged in a randomized complete block design (RCBD) with three replications. The experiment consisted of ten treatments as follows: (T1) control without the application of rock phosphate (RP), synthetic fertilizer and inoculum; (T2) inoculated with GM; (T3) inoculated with RI; (T4) inoculated with KV; (T5) inoculated with GM + KV; (T6) inoculated with RI + KV; (T7) inoculated with GM + KV plus application of rock phosphate; (T8) inoculated with RI + KV plus application of rock phosphate; (T9) rock phosphate only, without inoculation and; (T10) synthetic fertilizer. The RP applied to the plants amounted to 625 kg ha^−1^ while the chemical fertilizers (15 − 15 − 15) amounted to 156.25 kg ha^−1^. Plants were grown in pots containing 17 kg of non-sterilized soil. The Jerusalem artichoke genotype HEL65 was obtained from the Faculty of Agriculture, Khon Kaen University, Thailand. Seeding preparations were carried out according to Sennoi *et al*.^22^. For the mycorrhizal treatments, approximately 20 g of inoculum (25 spores g^−1^ soil) was added to the soil in the vicinity of the roots of Jerusalem artichoke immediately after transplanting. Bacterial application was performed by using a syringe, in which 10 mL of 10^9^ CFU mL^−1^ bacterial cell suspension was inoculated in the vicinity of the roots of the seedlings.

### Plant performance

Plant growth was assessed 75 days after transplanting. Plant height was measured, and dry weight of leaves, stems, roots and tubers was recorded after drying in an oven at 80 °C for 3 days. Leaf area (LA) was measured using a leaf area meter (Li-3100C Area meter). SPAD chlorophyll meter reading (SCMR) value of plant leaves was recorded using a chlorophyll meter SPAD-502 plus. The number of tubers was counted, and tuber fresh weight determined. In addition, plant root growth data (length, surface area volume, diameter, specific root length) were assessed with an Epson scanner V700 PHOTO meter and measured automatically using a WINRHIZO Pro2004a (REGENT Instruments Inc., QC, Canada). The determination of tuber inulin was done following a slight modification of the method described by Saengkanuk *et al*.^23^.

### Determination of PSB numbers

The spread-plate technique was performed to estimate PSB numbers in the rhizosphere. Rhizosphere soil was collected by uprooting and the soil adhering to the roots was serially diluted; 0.1 mL aliquots of the appropriate dilution were spread on Pikovskaya’s medium plates. The plates were incubated at 30 °C for 48 h. Colonies showing a clear zone indicative of tri-calcium phosphate dissolution were counted and displayed as log CFU mL^−1^.

### AMF colonization and spore numbers

Root samples were carefully washed by tap water and soaked in 10% KOH at 90 °C for 15 min, rinsed with tap water again, acidified in 1% HCl overnight, and then stained with 0.05% trypan blue in lacto-glycerol, according to Koske and Gemma^24^. The stained root segments (0.5–1 cm long) from each treatment were investigated for AMF root colonization intensity using the Trouvelot’s method^25^. The total amount of AMF spores in the soil was determined by the sucrose centrifugation method described by Daniels and Skipper^26^.

### Statistical analysis

Data are reported as means ± SE, based on three replicates. Data were subjected to one-way analysis of variance (ANOVA). For IAA production we excluded the strains that were negative for the one-way ANOVA, in order to comply with the demands for homogeneity of variances. The least significant difference (LSD) value test was applied to test for significant differences of the treatment mean at P < 0.05. Correlation between parameters was calculated by Pearson’s correlation coefficient and evaluated at the P < 0.05 significance level. All statistical analyses were performed using Statistix 8.0 software.

## Results

### Identification and characterization of PSB isolates

Eleven bacterial colonies exhibited distinct phosphate solubilizing activity (Table 1). Phylogenic analysis revealed that these 11 phosphate-solubilizing isolates belonged to six bacterial groups, viz. *Burkholderia tropica*, *Pseudomonas aeruginosa* (four strains)*, Achromobacter xylosoxidans*, *Ochrobactrum pseudogrignonense, Klebsiella variicola* and *Sphingobacterium thalpophilum* (three strains). Subsequently bacteria will be designated by their generic name only.

**Table 1:** PSB isolate (GenBank accession number), nearest Blast match (with percentage similarity), Phosphate solubilization index (PSI), solubilization of phosphate, pH of medium, types of organic acids produced, and IAA production by PSB isolates cultured in Pikovskaya’s broth medium containing 5% tri-calcium phosphate.

PSB Isolate (GenBank accession No.)	Nearest taxon	PSIs	^a^Amounts of dissolved P (µg mL^−1^)	pH of medium	Types of organic acid	IAA production (µg mL^−1^)
LC373005	*Sphingobacterium thalpophilum* (93%)	1.04	477 e	5.11 b	G, O, DL-M	negative
LC373006	*Klebsiella variicola* (95%)	1.73	371 h	4.38 d	G, O, L, A,	5.5 a
LC373008	*Sphingobacterium thalpophilum* (93%)	1.22	458 g	5.17 b	DL-M, A, L	2.8 b
LC373004	*Sphingobacterium thalpophilum* (93%)	2.10	470 f	5.12 b	G, O, DL-M	0.7 d
LC373003	*Ochrobactrum pseudogrignonense* (92%)	1.08	327 i	5.46 a	G, O, DL-M	negative
LC372998	*Burkholderia tropica* (97%)	1.84	502 c	4.26 d	G	0.1 e
LC373000	*Achromobacter xylosoxidans* (98%)	1.94	458 g	4.66 c	G, T, L, A, DL-M	negative
LC373007	*Pseudomonas aeruginosa* (98%)	1.17	526 a	4.20 d	G, T, L, A, DL-M	2.6 b
LC373002	*Pseudomonas aeruginosa* (98%)	1.30	489 d	4.68 c	G, T, L, DL-M	1.0 d
LC373001	*Pseudomonas aeruginosa* (98%)	1.10	489 d	4.72 c	G, T, L, DL-M	1.6 c
LC372999	*Pseudomonas aeruginosa* (98%)	2.00	520 b	4.68 c	G, DL-M	3.0 b
F-test		ns	**	**		**
% CV		34.94	0.20	2.65		13.11

^a^Liquid cultures were assayed after 72 h. G, gluconic acid; O, oxalic acid; DL-M, DL-marlic acid; L, lactic acid; A, acetic acid; T, tartaric acid. Values with a different letter within the same column are significantly different at P ≤ 0.05 by least significant difference (LSD). **Significant at P ≤ 0.01; *Significant at P ≤ 0.05; ns, non-significant.

Measurements of PSI, soluble phosphorus, IAA production, type of organic acid produced, and pH changes in the medium are also shown in Table 1. Values of PSI ranged between 1.10 and 2.10, however, there were no significant differences between isolates. The soluble phosphorus concentration in the medium ranged between 327 to 526 µg mL^−1^, and varied among the strains. Highest amounts of soluble P were produced by *Burkholderia* and two strains of *Pseudomonas*, the lowest amounts by *Ochrobactrum* and *Klebsiella*. All strains acidified the medium, and final pH ranged from 4.20 to 5.46, from an initial pH of 7.0. The strongest acidification was caused by *Pseudomonas* (one strain), *Burkholderia* and *Klebsiella*, while the lowest acidification was caused by *Ochrobactrum*. The phosphate solubilization in the liquid medium was not correlated with pH (r = -0.47 ; P = 0.14). The highest IAA production was found for *Klebsiella*, while one strain of *Sphingobacterium*, *Ochrobactrum* and *Achromobacter* did not produce IAA. HPLC analysis showed the presence of six organic acids, viz., gluconic, oxalic, DL-marlic, lactic, acetic, and tartaric acid. Gluconic acid was produced by ten isolates, only one strain of *Sphingobacterium* did not produce it. All but one strain produced at least two organic acids and two strains produced five organic acids. On the basis of high IAA production and strong acidifying effect (despite a relatively low amount of P that was solubilized), we selected the *Klebsiella* strain for the subsequent experiment to investigate the effect of co-inoculation with AMF on growth promotion and tuber inulin content of Jerusalem artichoke.

### Performance of Jerusalem artichoke

**Figure 1:**
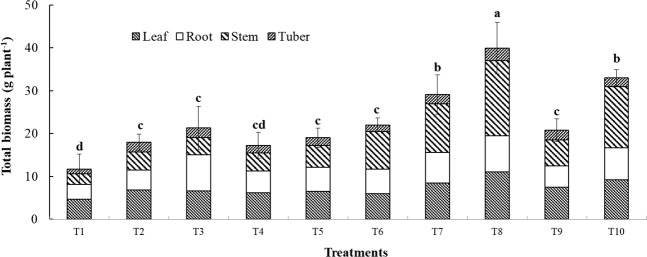
Effects of co-inoculation of AMF and PSB and fertilizer application on dry mass production of Jerusalem artichoke. Different letters indicate significant differences at P < 0.05 by LSD. Treatment means are the average of three replications. Treatments: (T1) Control, (T2) inoculated with GM, (T3) inoculated with RI, (T4) inoculated with KV, (T5) inoculated with GM + KV, (T6) inoculated with RI + KV, (T7) inoculated with GM + KV + RP, (T8) inoculated with RI + KV + RP, (T9) RP, (T10) synthetic fertilizer.

All treatments, apart from single inoculation with *Klebsiella* T4) resulted in increased dry weight of Jerusalem artichoke compared to the control (T1). Compared to the treatment with synthetic fertilizer only (T10), the treatment with *Klebsiella*, *Rhizophagus* and rock phosphate (T8) resulted in higher biomass; the comparable treatment where *Glomus* rather than *Rhizophagus* was used (T7), resulted in plants that were of same biomass as the fertilized control. Treatments with only a mycorrhizal fungus or with the mycorrhizal fungus plus *Klebsiella* in the absence of rock phosphate, or where rock phosphate was added in the absence of inoculum resulted in plants that had less biomass than the fertilized treatment (Fig. 1).

The results for plant height, leaf area, SCMR values and tuber characteristics are provided in Table 2. Plant height and leaf area were lowest in the unfertilized control (T1) and highest in the treatment with *Klebsiella*, *Rhizophagus* plus rock phosphate (T8). SCMR values did not show significant differences between treatments. Tuber fresh weight was contrary lowest in the unfertilized control (T1) and highest in T8. Tuber fresh weight was significantly higher than the unfertilized control in all treatments. Plant biomass was significantly positively correlated with plant height and the number of tubers (P < 0.01 in all cases), and marginally so with tuber fresh weight (P = 0.058). Tuber number and mass of individual tubers were (almost) significantly negatively correlated (P = 0.0503).

**Table 2:** Plant growth parameters of Jerusalem artichoke treated by dual cultured AMF and PSB.

^a^Treatments	Height (cm)	Leaf area (cm^2^)	SCMR values	TFW (g)	NT	WIT (tuber g−^1^)
T1	31.8 d	653.5 d	31.0	4.8 e	3.7 c	1.3 b
T2	66.6 abc	982.4 c	34.4	17.2 a	6.7 bc	2.6 a
T3	55.7 abcd	1,043.1 c	32.7	12.5 bcd	8.2 bc	1.5 b
T4	51.3 cd	1,028.7 c	35.1	10.9 cd	11.3 b	1.0 b
T5	52.8 bcd	1,104.7 bc	31.7	11.4 cd	10.8 b	1.1 b
T6	72.2 abc	949.2 c	33.8	9.3 d	10.4 b	1.0 b
T7	78.8 a	1,132.8 bc	33.2	13.2 bc	18.9 a	0.7 b
T8	79.5 a	1,423.0 a	35.5	17.4 a	22.7 a	0.8 b
T9	67.2 abc	1,022.0 c	34.2	15.3 ab	5 bc	3.4 a
T10	75.7 ab	1,350.9 ab	31.3	14.1 abc	10.4 b	1.4 b
F-test	*	*	ns	**	**	**
% CV	22.18	15.39	10.07	16.62	35.67	34.72

The mean for height, SPAD chlorophyll meter reading (SCMR), leaf area (LA), tuber fresh weight (TFW), number of tubers (NT) and weight of individual tuber (WIT) were evaluated at 75 days after transplanting under pots conditions.

**Significant at P < 0.01; *Significant at P < 0.05 and ns, non-significant. Data are the means of three replications. Within each column, values with the same lower-case letter are not significantly different at P < 0.05. ^a^For treatments see Materials and Methods.

**Figure 2:**
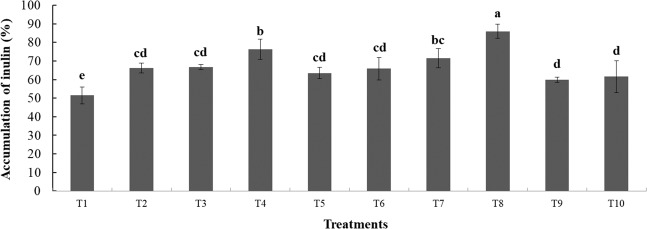
Tuber inulin accumulation of Jerusalem artichoke variety HEL65. Different letters indicate significant differences at P < 0.05 by LSD. Treatment means are the average of three replications. Treatments: (T1) Control, (T2) inoculated with GM, (T3) inoculated with RI, (T4) inoculated with KV, (T5) inoculated with GM + KV, (T6) inoculated with RI + KV, (T7) inoculated with GM + KV + RP, (T8) inoculated with RI + KV + RP, (T9) RP, (T10) synthetic fertilizer.

Accumulation of inulin in the tubers of Jerusalem artichoke was lowest in the unfertilized control and highest in T8, the treatment with *Klebsiella*, *Rhizophagus* and rock phosphate. The treatment with only *Klebsiella* (T4) and with the combination of *Klebsiella*, *Glomus* and rock phosphate (T7) had significantly higher inulin content than the fertilized control and the treatment with only rock phosphate (T10 and T9 respectively; Fig. 2), indicating that biological interactions rather than fertilizer promoted inulin content.

Root length, root surface area and root volume of plants were significantly correlated with plant biomass (data not shown). Root traits showed the highest value in T8 and the lowest value in the unfertilized control (T1; Table 3). Inoculation with *Klebsiella* and/or AMF in the absence of RP did not have a significant effect on root diameter (Table 3).

**Table 3:** Inoculation effect on root length, surface area, volume and root diameter of Jerusalem artichoke, evaluated at 75 days after transplanting under pots condition.

^a^Treatments	Length (m)	Surface area (cm^2^)	Volume (cm^3^)	Diameter (mm)
T1	23.082 d	2046 e	14.5 f	0.29
T2	50.161 c	4238 cd	28.6 cdef	0.27
T3	87.751 a	6792 ab	42.9 abc	0.25
T4	42.875 cd	3527 cde	24.8 def	0.26
T5	88.484 a	7554 a	51.4 ab	0.27
T6	56.009 c	5164 bc	38.0 abcde	0.29
T7	80.638 ab	6310 ab	39.5 abcd	0.25
T8	93.265 a	7846 a	54.0 a	0.27
T9	37.233 cd	3125 de	22.4 ef	0.27
T10	57.705 bc	5053 bcd	35.3 bcde	0.28
F-test	**	**	**	ns
% CV	22.7	22.81	27.08	8.29

**Significant at P ≤ 0.01; ns, non-significant. Data are the means ± SE (n = 3). Different lower-case letters indicate significant differences (P < 0.05) using Fisher’s Least Significant Difference (LSD). ^a^For treatments see Materials and Methods.

### Abundance of Phosphate-solubilizing bacteria and AMF

Mycorrhizal colonization (Table 4) was lowest in the unfertilized control (T1), followed by the fertilized treatments with rock phosphate and synthetic P fertilizer (T9 and T10). It was also significantly lower in the treatment with *Glomus* (T2) only than in all other treatments with *Klebsiella* and/or AMF. However, spore numbers of AMF in T2 were not particularly low. Spore numbers were lowest in the unfertilized control and in both fertilizer treatments (T1, T9, T10). Abundance of PSB was highest in the treatments where *Klebsiella* had been added together with AMF and rock phosphate (T4, T7, T8). Addition of rock phosphate (T9) did not increase abundance of PSB compared to the treatment with synthetic P fertilizer (T10), however in both treatments PSB abundance was higher than in the unfertilized control (T1; Table 4).

**Table 4:** Root colonization, abundance of AMF spores, and abundance of PSB in soil.

^a^Treatments	AMF colonization rate (%)	PSB population (log CFU g^−1^)	AMF spores (spores g soil^−1^)
T1	17.33 e	0.47 f	0.93 e
T2	30.33 d	2.10 de	5.27 d
T3	55.67 c	2.70 d	6.20 cd
T4	55.00 c	5.57 ab	1.27 e
T5	66.93 b	4.03 c	7.20 bc
T6	72.83 b	5.20 b	7.40 b
T7	88.80 a	5.57 ab	6.20 cd
T8	91.60 a	6.00 a	8.77 a
T9	29.33 d	1.67 e	1.73 e
T10	25.90 d	1.50 e	1.87 e
F-test	**	**	**
% CV	9.16	13.21	13.20

**Significant at P < 0.01. Data are the means of three replications. Within each column, values with the same lower-case letter are not significantly different at P < 0.05. ^a^For treatments see Materials and Methods.

Mycorrhizal colonization and spore numbers were significantly positively correlated with root volume, root surface area and root length, but not with root diameter. The abundance of PSB was also significantly positively correlated with the three root traits (Table 5).

**Table 5:** Correlation between the percentage of AMF colonization, PSB population, and AMF spore density in soil and plant root growth (volume, surface area, length and diameter).

Variable	Root volume	Root surface area	Root length	Root diameter
AMF colonization	0.64**	0.67**	0.68**	−0.21 ns
PSB population	0.52**	0.52**	0.51**	−0.14 ns
Spore density	0.74**	0.78**	0.77**	−0.12 ns

**and ns Significant at P ≤ 0.01 and non-significant probability levels, respectively.

## Discussion

The 11 strains with phosphate-solubilizing capacity belonged to six different genera. The ability to solubilize P for these genera has been reported before. Their PSIs ranged from 1.04 to 2.10 in our study, with no significant differences between isolates. Our findings are similar to results reported by Teymouri *et al*.^27^. The P solubilization resulted in a drop in the pH of the broth media. Species that showed stronger acidification tended to release more P in the medium, however, there were exceptions to this rule, as *Klebsiella* had a strong pH effect but did release comparatively little P in the medium. Earlier studies reported a significant negative correlation between the pH of the medium and soluble phosphate^28,29^. The pH decline may be due to release of protons, while proton extrusion together with the production of carboxylates may have caused the solubilization of phosphate. HPLC analysis detected six organic acids, gluconic acid, oxalic acid, DL-marlic acid, lactic acid, acetic acid and tartaric acid. Our results are largely in agreement with findings of earlier studies by Wei *et al*.^30^ and Li *et al*.^31^. Gluconic acid was the most frequently produced (by 10 out of 11 strains) organic acid, similar to the findings of Chen *et al*.^32^. Contrastingly, studies by Marra *et al*.^33^ and Surapat *et al*.^19^ reported propionic acid as the major organic acid, with the following order propionic> gluconic> tartaric> malic acid.

Next to their ability to solubilize several PSB have also been reported to produce plant hormones such as IAA, the most common plant hormone of the class of auxins. Such bacteria are also known as plant-growth promoting rhizobacteria (PGPR). Many important plant–microbial interactions center on the production of auxins, with IAA being the main plant auxin. IAA is responsible for the division, expansion and differentiation of plant cells and tissues and also stimulates root elongation^34^. Eight out of our 11 strains studied were positive for IAA production. Previous studies had already documented that many strains of PSB can produce high IAA values, belonging to *Aeromonas* and *Burkholderia*^35^, *Azotobacter*^36^, *Bacillus*^37^, *Enterobacter*^38^, *Achromobacter*^39^ and *Rhizobium*^40^. In our study *Klebsiella variicola* produced the highest amount of IAA. Currently, there are only a few reports on its plant-growth promoting activity^41^.

Interactions between mycorrhizal fungi and bacteria (PSB, PGPR, rhizobia) have often described as synergistic, however it has not always been made explicit how synergy had to be operationalized. Here we follow Neetu *et al*.^42^. and Tahat *et al*.^43^ who distinguished between additive and synergistic effects. Synergistic effects occur if in a two-factorial experiment, where the fungi and the bacteria are manipulated experimentally, the interaction term is significant. If the interaction term is not significant, the effects are additive. Under that criterion several instances of synergy actually turn out to be mere additive effects based on the independent action of both microbial groups. Synergistic effects can then be positive, in case the effect is more-than-additive, or negative when the effect is less-then-additive. In order to test for possibly synergy, we additionally analyzed the results of part of the experiment (treatments T1-T6) as a two factor experiment with *Klebsiella* (present or absent) and AMF (two species plus a control). The results of that analysis are shown in Table 6. Inspection of that table shows that many AMF × PSB interactions are indeed significant. However, in all cases effects are less than additive, indicating that the combination of both AMF and PSB in the absence of an external sparingly soluble P sources does not yield synergy. Unfortunately, we did not include experiments with only *Klebsiella* or only one AMF species in the presence of rock phosphate, so the possible occurrence of synergistic effects after addition of a sparingly soluble P sources awaits further studies. Considering the high inulin content in the tripartite combination of rock phosphate, AMF and PSB, where each of the individual factors showed only marginal effects on inulin production, we hypothesize that in that case synergy may likely occur. However, even if effects are additive or only somewhat less-than-additive, clear benefits of co-inoculation are evident in our study. Our conclusion that positive synergy was absent in our system is consistent with a meta-analysis by Larimer *et al*.^44^ who noted that the interaction between rhizobia and AMF also did not show synergistic effects.

**Table 6:** Two-way ANOVA on plant data to test for synergistic effects between AMF and PSB.

Variable	Height	Leaf area	SCMR	TFW	NT	WIT	Biomass	Inulin content	Root length	Root surface	Root volume	Root diameter
AMF (df = 2)	6.92 (*)	5.34 (*)	0.01 (ns)	21.39 (**)	0.83 (ns)	9.26 (**)	30.24 (**)	0.46 (ns)	23.21 (**)	19.83 (**)	14.32 (**)	0.06 (ns)
PSB (df = 1)	2.03 (ns)	6.47 (*)	0.39 (ns)	1.42 (ns)	15.46 (**)	31.26 (**)	7.64 (*)	12.28 (**)	2.84 (ns)	5.02 (*)	6.73 (*)	1.03 (ns)
AMFxPSB (df = 2)	4.15 (*)	6.57 (*)	2.26 (ns)	20.72 (**)	1.81 (ns)	7.04 (*)	1.27 (ns)	19.37 (**)	16.16 (**)	9.36 (**)	4.90 (*)	7.74 (**)

*, **and ns: significant at P < 0.05, P < 0.01 and non-significant probability levels, respectively.

In the presence of rock phosphate, the dual inoculation with PSB and AMF (T8) enhanced almost all plant growth parameters (except chlorophyll readings and weight of individual tubers). Tuber number and weight of individual tubers were (almost) significantly negatively correlated and maximizing both parameters is therefore impossible. However, both tuber number and tuber mean weight contribute to total tuber mass per plant and so increased understanding how selection for both traits would result in trade-offs is imperative.

The beneficial effects of dual inoculation on plant performance and the highest abundance of PSB in the treatments where *Klebsiella* was inoculated would suggest successful establishment and survival of this bacterium in the rhizosphere of Jerusalem artichoke. However, we did not sequence the PSB that were isolated, and so cannot conclude the possibility that in some treatments, especially the dual-inoculation treatments, rhizosphere modification by AMF also increased the abundance of other PSB that *Klebsiella*. Changes in the bacterial community in the rhizosphere as a result of mycorrhizal inoculum have been reported before^45,46^.

In our experiment we selected a strain of *Klebsiella* because it both enhanced phosphate solubilization, through acidification of the environment by exuding organic acids, and because it showed high productivity of IAA, which could subsequently modify plant root growth. Attribution of causality to either enhanced rock phosphate dissolution and P uptake from soluble P after dual inoculation (T8) or root modification is therefore not possible. Positive effects of PSB (or PGPR) through root modification have been reported by Kavatagi and Lakshman^47^ who noted enhanced root growth after inoculation with AMF and PSB. Their data also suggest higher specific root length after inoculation, consistent with fungal and / or bacterial impacts on root morphology. Our findings are also consistent with results reported by Pindi *et al*.^34^ who documented high IAA production and enhanced growth performance of a cotton variety when inoculated with a strain of *Bacillus* that both showed phosphate-solubilizing activity and enhanced IAA production. Beneficial effects of PSB have also been described for a suite of further crops by Shankar *et al*.^48^ and Singh *et al*.^49^.

While synthetic fertilizer (T10) resulted in larger plants than the application of rock phosphate (T9), we noted no differential effect of both P sources on plant inulin content. This result fits with an increasing body of papers that show that beneficial soil microbes, especially AMF, do not only enhance plant performance but also the concentration of useful secondary compounds^50^. Dual inoculation did enhance the effect of rock phosphate application, and dual inoculation could therefore replace expensive synthetic fertilizers by cheaper sources of P fertilizer. While we did not test for effects of dual inoculation in the presence of synthetic fertilizer, because costs for that practice would likely be prohibitive for Thai farmers, we noted that Neetu *et al*.^42^ described growth enhancement of flax (*Linum usitatissimum*) in the presence of PSB (*Pseudomonas fluorescens*) and AMF, allowing reduction of the amount of synthetic fertilizers that have to be applied.

## Conclusions

Our investigation revealed beneficial effects of dual inoculation of a PSB strain (*Klebsiella variicola*) and AMF (especially *Rhizophagus intraradices*), when combined with addition of rock phosphate, in the growth promotion and inulin production of Jerusalem artichoke. The *Klebsiella* strain produced both IAA and organic acids that resulted in the acidification and dissolution of rock phosphate in culture conditions, and we suppose that this event may occur in the soil. The available P was subsequently taken up and transported to the root of Jerusalem artichoke by AMF. Therefore, dual inoculation may be a promising strategy to both reduce expensive synthetic fertilizers and to enhance inulin production. In order to verify these effects, field trials should be undertaken as the next step, before these plant-growth promoting microorganisms can be applied by farmers for the sustainable production of Jerusalem artichoke in the future.
